# Induction of pro-inflammatory genes by serum amyloid A1 in human amnion fibroblasts

**DOI:** 10.1038/s41598-017-00782-9

**Published:** 2017-04-06

**Authors:** Wenjiao Li, Wangsheng Wang, Rujuan Zuo, Chao Liu, Qun Shu, Hao Ying, Kang Sun

**Affiliations:** 1 Center for Reproductive Medicine, Ren Ji Hospital, School of Medicine, Shanghai Jiao Tong University, Shanghai, P.R. China; 2 Shanghai Key Laboratory for Assisted Reproduction and Reproductive Genetics, Shanghai, P.R. China; 3 Department of Obstetrics and Gynecology, Zhongshan Hospital, Fudan University, Shanghai, P.R. China; 4 Shanghai First Maternity and Infant Hospital, Tongji University School of Medicine, Shanghai, P.R. China

## Abstract

Serum amyloid A1 (SAA1) is an acute response protein, which is mainly produced by the liver, during infection. However, it remains unknown whether SAA1 can be produced in human fetal membranes where it is able to elicit events pertinent to labor initiation. We demonstrated that SAA1 was expressed in the fibroblasts and epithelium of the amnion and the trophoblasts of the chorion. Further study in human amnion fibroblasts showed that SAA1 production was augmented by interleukin-1β (IL-1β) and cortisol alone and synergistically, and SAA1 in turn induced the expression of IL-1β, interleukin-6 (IL-6), cyclooxygenase-2 (COX-2) and PGE2 production. These effects of SAA1 were mediated through activation of the NF-κB, p38 and ERK1/2 pathways via the toll-like receptor 4 (TLR4). Inhibition of TLR4 attenuated not only SAA1-induced activation of NF-κB, p38 and ERK1/2 but also increases in IL-1β, IL-6 and COX-2 expression. Moreover, SAA1 expression was increased in human amnion tissue following spontaneous labor. In conclusion, this study has demonstrated for the first time that SAA1 can be produced in human fetal membranes, which can be greatly induced in the presence of proinflammatory cytokines and glucocorticoids thereby producing effects associated with parturition.

## Introduction

Serum amyloid A1 (SAA1) is an inducible acute phase protein in response to injury, infection and inflammation^1, 2^. During the acute phase of the inflammatory response, the liver secretes a large amount of SAA1 resulting in a marked increase in SAA1 level in the blood^3^. SAA1 is not only the major component of amyloid A deposit in tissues undergoing prolonged inflammation^4^, but can also elicit a number of actions such as induction of immune cell migration^5, 6^, stimulation of cytokine/chemokine production^7–10^ and mediation of bacteria uptake by neutrophils^11^, at the inflammation site. Several receptors have been identified to mediate the actions of SAA1 including the N-formyl peptide receptor 1 (FPR1)^12^, toll-like receptor 2 (TLR2)^13^ and toll-like receptor 4 (TRL4)^14^. Intracellularly, it has been reported that SAA1 is capable of activation of the nuclear factor kappa-light-chain-enhancer of activated B cells (NF-κB) and mitogen-activated protein kinases (MAPKs) pathways^15–17^. Although the hepatocyte is known to be the major site of SAA1 production, a number of other cell types including tumor cells^18, 19^ and placental trophoblast cell lines^4, 20^ have been documented to express SAA1. However, it remains unknown whether the human fetal membranes, a source of a number of crucial factors involved in the initiation of parturition^21, 22^, are also able to produce SAA1.

Among the factors produced by the fetal membranes, proinflammatory cytokines and prostaglandin E2 (PGE2) are particularly important in terms of labor initiation under both infection and non-infection-induced inflammation^23–25^. While infection-induced chorioamnionitis is a common cause of preterm birth, non-infection-induced inflammation of the fetal membranes, the so-called sterile inflammation, is also recognized to be essential for both term and preterm birth^24^. Proinflammatory cytokines produced in the fetal membranes not only reinforce local inflammatory responses leading to the rupture of the fetal membranes^25^, but also enhance the synthesis of PGE2, a potent uterotonic hormone, locally in the fetal membranes^25^. Indeed, increased interleukin-1β (IL-1β), interleukin-6 (IL-6) and PGE2 production are associated not only with infection-induced preterm labor but also with term labor without confirmed infection in the fetal membranes^24, 26^. The mesenchymal fibroblasts in the amnion layer of the fetal membranes are particularly important in terms of synthesis of these parturition-related factors. Amnion fibroblasts are not only a major source for PGE2 towards the end of gestation^27, 28^ but they are also capable of producing proinflammatory cytokines and regenerating cortisol^28–30^. Interestingly, glucocorticoids and proinflammatory cytokines have been reported to induce SAA1 expression synergistically in hepatocytes^31^. Given the aforementioned endocrine entity of amnion fibroblasts and the inflammation-inducing properties of SAA1, we hypothesize that the production of SAA1 in amnion fibroblasts may be under the paracrine or autocrine drive by cortisol and proinflammatory cytokines, and in turn SAA1 may regulate the expression of IL-1β, IL-6 and cyclooxygenase-2 (COX-2), the key enzyme involved in the production of PGE2, in amnion fibroblasts, thereby participating in the initiation of parturition.

In this study, we examined the local production of SAA1, the regulation of SAA1 production by IL-1β and cortisol, the effects of SAA1 on IL-1β, IL-6 and COX-2 expression in cultured primary human amnion fibroblasts as well as the effect of labor on the abundance of SAA1 in human amnion tissue. It is anticipated that elucidation of these processes will help further understand the mechanisms of both normal term and pathogen-induced preterm birth.

## Results

### Expression of SAA1 in human fetal membranes and amnion fibroblasts

**Figure 1:**
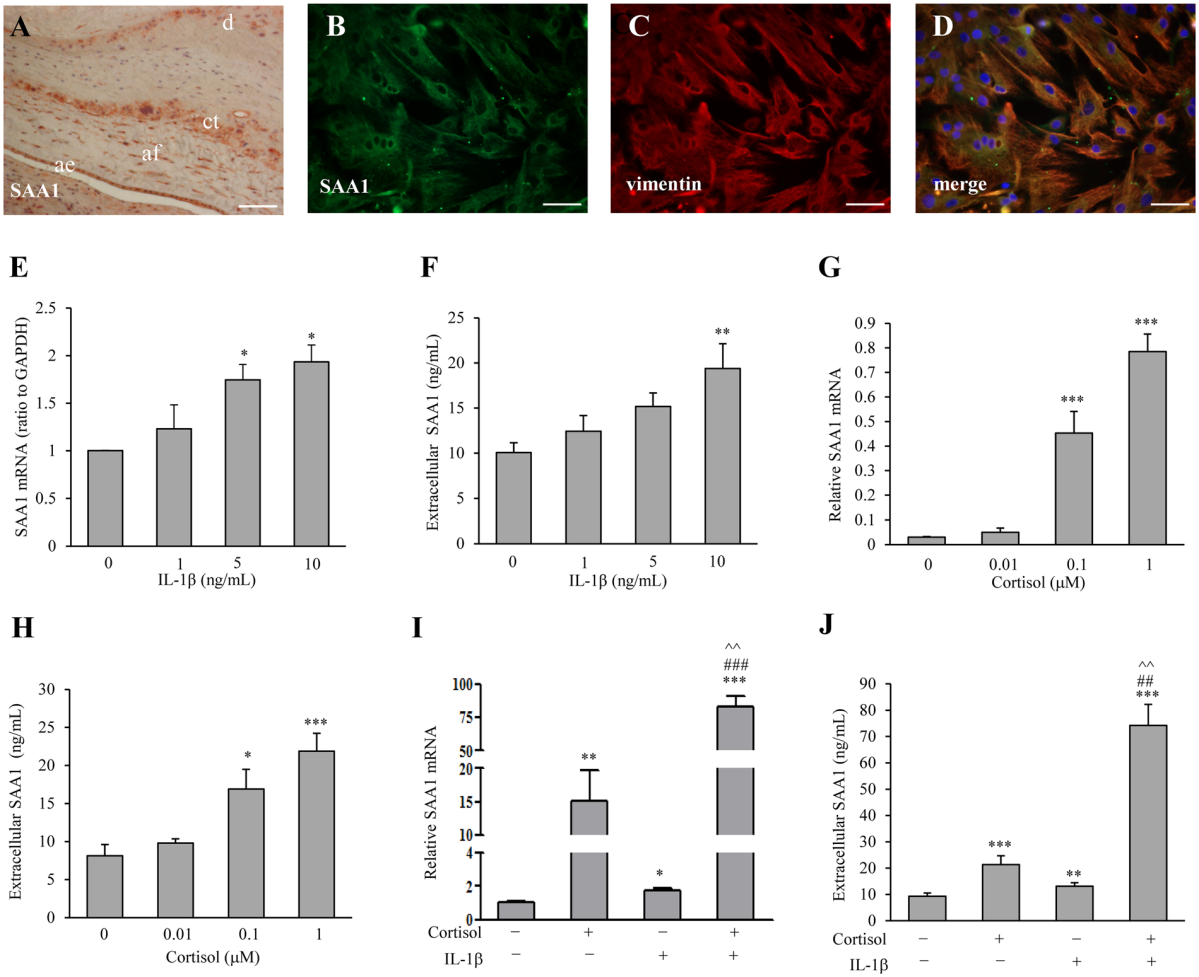
Expression of SAA1 in human fetal membranes and induction of SAA1 production by cortisol and IL-1β. **(A**) Immunohistochemical staining of SAA1 in human fetal membranes. ae: amnion epithelial cells; af: amnion fibroblasts; ct: chorionic trophoblasts; d: decidua. Scale bar, 200 µm. **(B–D**) Immunofluorescence staining of SAA1 (B, green) and vimentin (C, red), a marker of mesenchymal cells, in human amnion fibroblasts. Yellow color after merging B and C represents merged staining for SAA1 and vimentin (**D**). Nuclei were counterstained blue with DAPI. Scale bar, 100 µm. (**E–H**) The effect of IL-1β (1, 5 and 10 ng/mL, 24 hours) and cortisol (0.01, 0.1 and 1 µM, 24 hours) on SAA1 mRNA (**E** and **G**) and secreted SAA1 protein (**F** and **H**) in human amnion fibroblasts. n = 4. (**I,J**) The synergistical effect of IL-1β (10 ng/mL) and cortisol (1 µM) on SAA1 mRNA (**G**) and secreted SAA1 protein (**J**) in human amnion fibroblasts. n = 4. Data are the means ± SEM. Statistical analysis was performed with one-way ANOVA test (**E–J**). *P < 0.05, **P < 0.01, ***P < 0.001 vs. control (0), ^##^P < 0.01, ^###^P < 0.001 vs. IL-1β. ^^P < 0.01 vs. cortisol.

Immunohistochemical staining of human fetal membranes revealed that the staining for SAA1 was found in amnion epithelial and fibroblast cells as well as in chorionic trophoblasts (Fig. 1A). Immunofluorescent staining of cultured human amnion fibroblast cells showed the positive staining for SAA1 in the cytoplasm (Fig. 1B). The cells that were stained for SAA1 were also stained positive for vimentin, a mesenchymal cell marker (Fig. 1C and D). In addition to the presence of SAA1 protein, SAA1 mRNA was also expressed in amnion fibroblasts (Fig. 1E). Moreover, there was a basal SAA1 secretion in cultured amnion fibroblasts, which reached a concentration of approximately 10 ng/mL over 24-hour incubation period (Fig. 1F). Furthermore, either IL-1β (1, 5 and 10 ng/mL) or cortisol (0.01, 0.1 and 1 µM) treatment of amnion fibroblasts increased SAA1 mRNA and secretion in a concentration dependent manner with more pronounced increases observed with cortisol treatment (Fig. 1E–H). Moreover, combination of IL-1β (10 ng/mL) and cortisol (1 µM) treatments dramatically increased SAA1 mRNA and protein in a synergistic manner (Fig. 1I and J).

### Induction of IL-1β, IL-6 and COX-2 by SAA1 in human amnion fibroblasts

**Figure 2:**
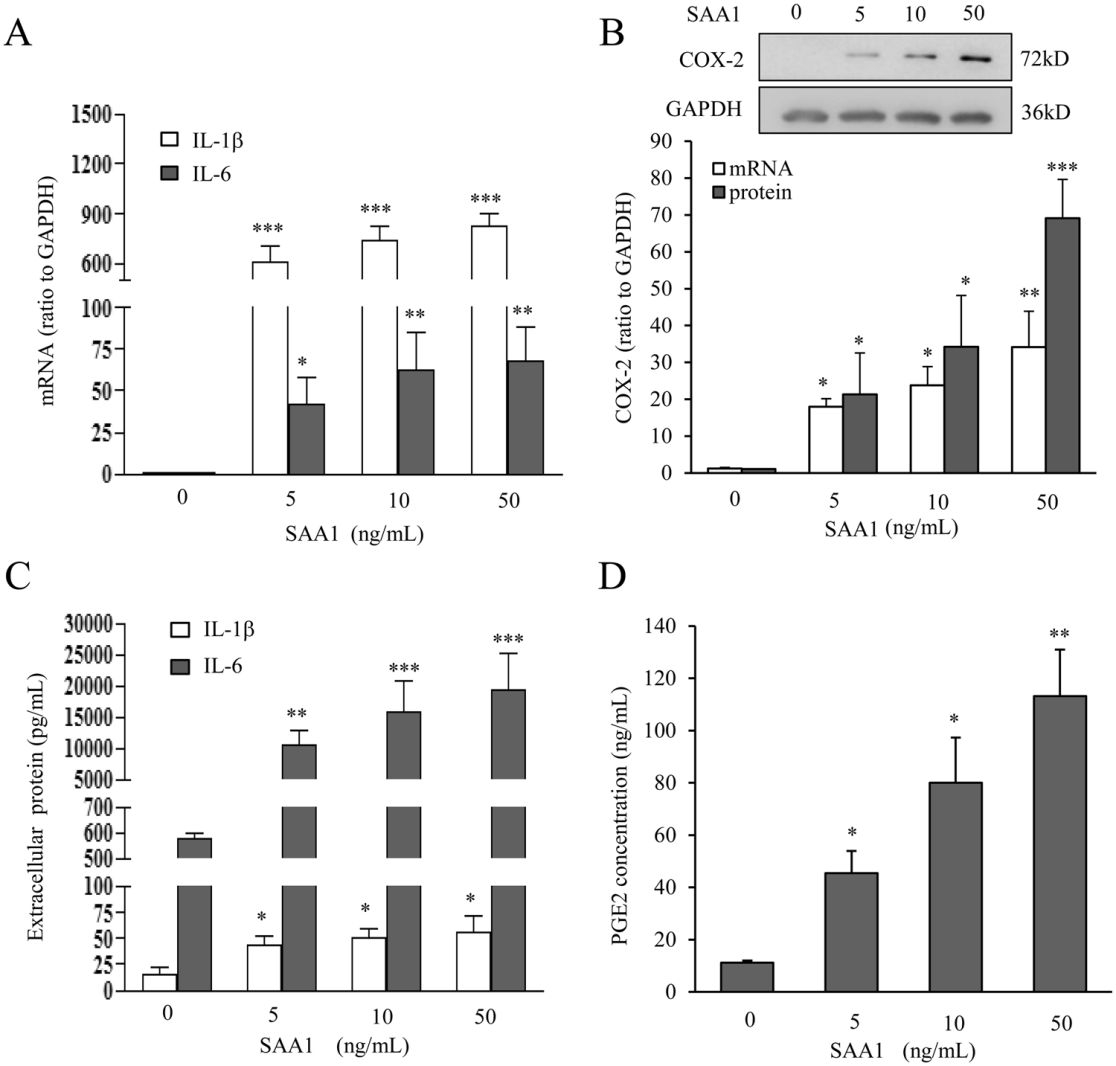
Induction of IL-1β, IL-6 and COX-2 by SAA1 in human amnion fibroblasts. (**A–D**) Effect of SAA1 (5, 10 and 50 ng/mL, 24 hours) on the amounts of IL-1β, IL-6 mRNA (A, n = 4), COX-2 mRNA and protein (**B**) n = 4), IL-1β and IL-6 secretion (C, n = 5), and PGE2 secretion (D, n = 3) in human amnion fibroblasts. Top panel of B is a representative immunoblot. Data are the means ± SEM. Statistical analysis was performed with one-way ANOVA test. *P < 0.05, **P < 0.01, ***P < 0.001 vs. control (0).

Treatment of amnion fibroblasts with SAA1 (5, 10 and 50 ng/mL, 24 hours) increased IL-1β, IL-6 and COX-2 mRNA and protein abundance in a concentration-dependent manner (Fig. 2A and B). Consistently, SAA1 treatment also increased IL-1β, IL-6 and PGE2 secretion in a concentration-dependent manner in amnion fibroblasts (Fig. 2C and D). To test whether these effects are ascribed to the trace amount of endotoxin in the preparation of recombinant SAA1, the cells were treated with 5 pg/mL endotoxin, which is equivalent to the amount of endotoxin contained in 50 ng of SAA1 according to the manual provided by the manufacturer. At this concentration, endotoxin affected neither IL-1β/IL-6 nor COX-2 mRNA expression (Supplementary Figure S2A and B) in amnion fibroblasts. To further rule out the possible effect of contaminating endotoxin, the cells were treated with SAA1 (10 ng/mL) or lipopolysaccharides (LPS) (5 ng/mL) in the presence of an endotoxin inhibitor polymyxin B (25 µg/mL). Polymyxin B only blocked the induction of IL-1β, IL-6 and COX-2 mRNA expression by LPS but not by SAA1 (Supplementary Figure S3A and B). These data suggest that SAA1 can induce the expression of pro-inflammatory genes pertinent to labor-initiation in human amnion fibroblasts and these effects are ascribed to the specific effects of SAA1 rather than to the trace amount of endotoxin contained in the recombinant SAA1 used.

### Roles of MAPKs and NF-κB in the induction of IL-1β, IL-6 and COX-2 by SAA1 in human amnion fibroblasts

**Figure 3:**
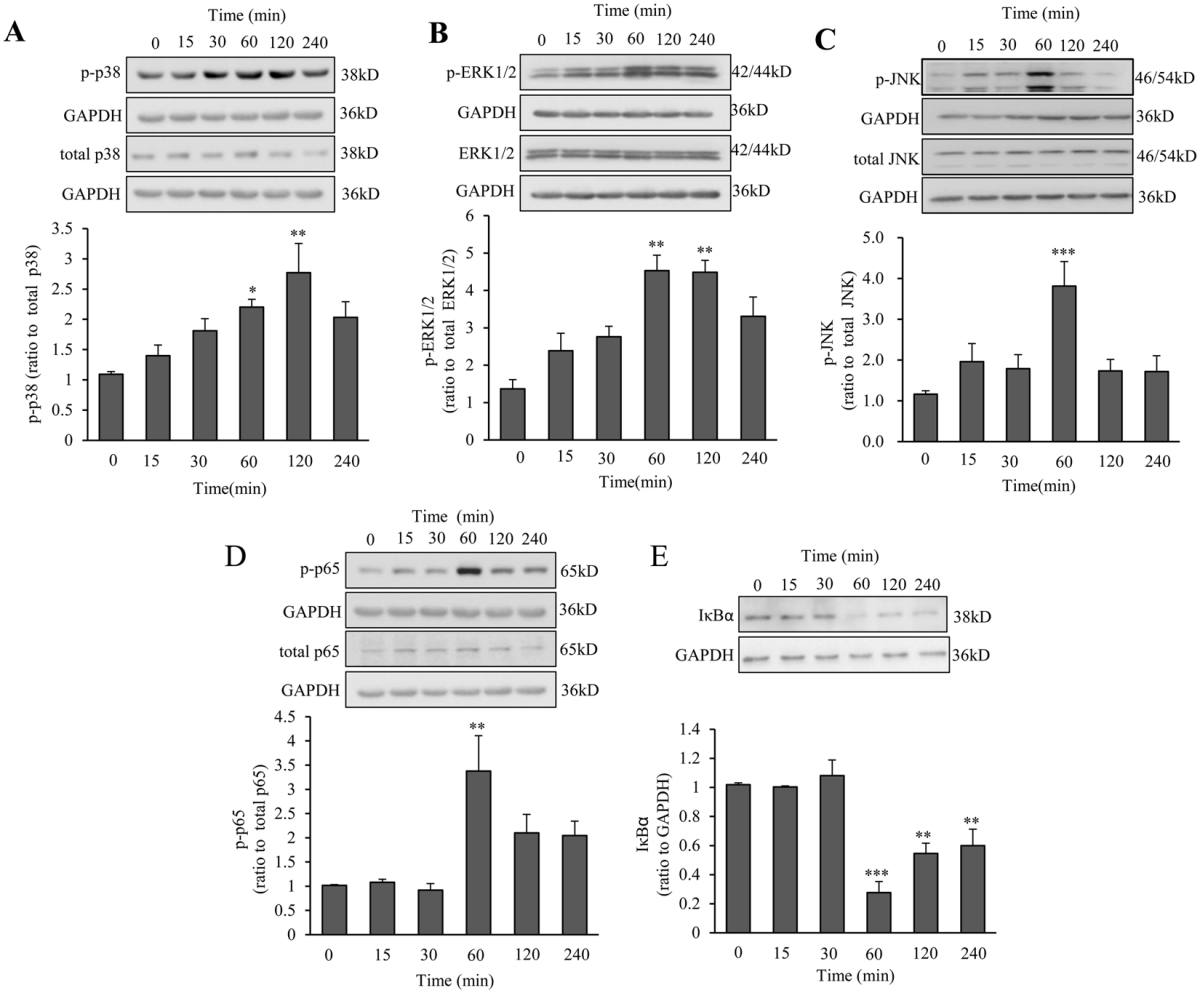
Effect of SAA1 on the activation of MAPKs and NF-κB in human amnion fibroblasts. **(A–C**) Effect of SAA1 (10 ng/mL; 15, 30, 60, 120 and 240 min) on the phosphorylation of p38 (**A**, n = 4), ERK1/2 (**B**, n = 3) and JNK (**C**, n = 4) in human amnion fibroblasts. **(D,E**) Effect of SAA1 (10 ng/mL; 15, 30, 60,120 and 240 min) on the abundance of phosphorylated p65 (**D**, n = 4) and total IκBα (**E**, n = 3) in human amnion fibroblasts. Top panels are the representative immunoblots. Data are the means ± SEM. Statistical analysis was performed with one-way ANOVA test. *P < 0.05, **P < 0.01, ***P < 0.001 vs. control (0).

**Figure 4:**
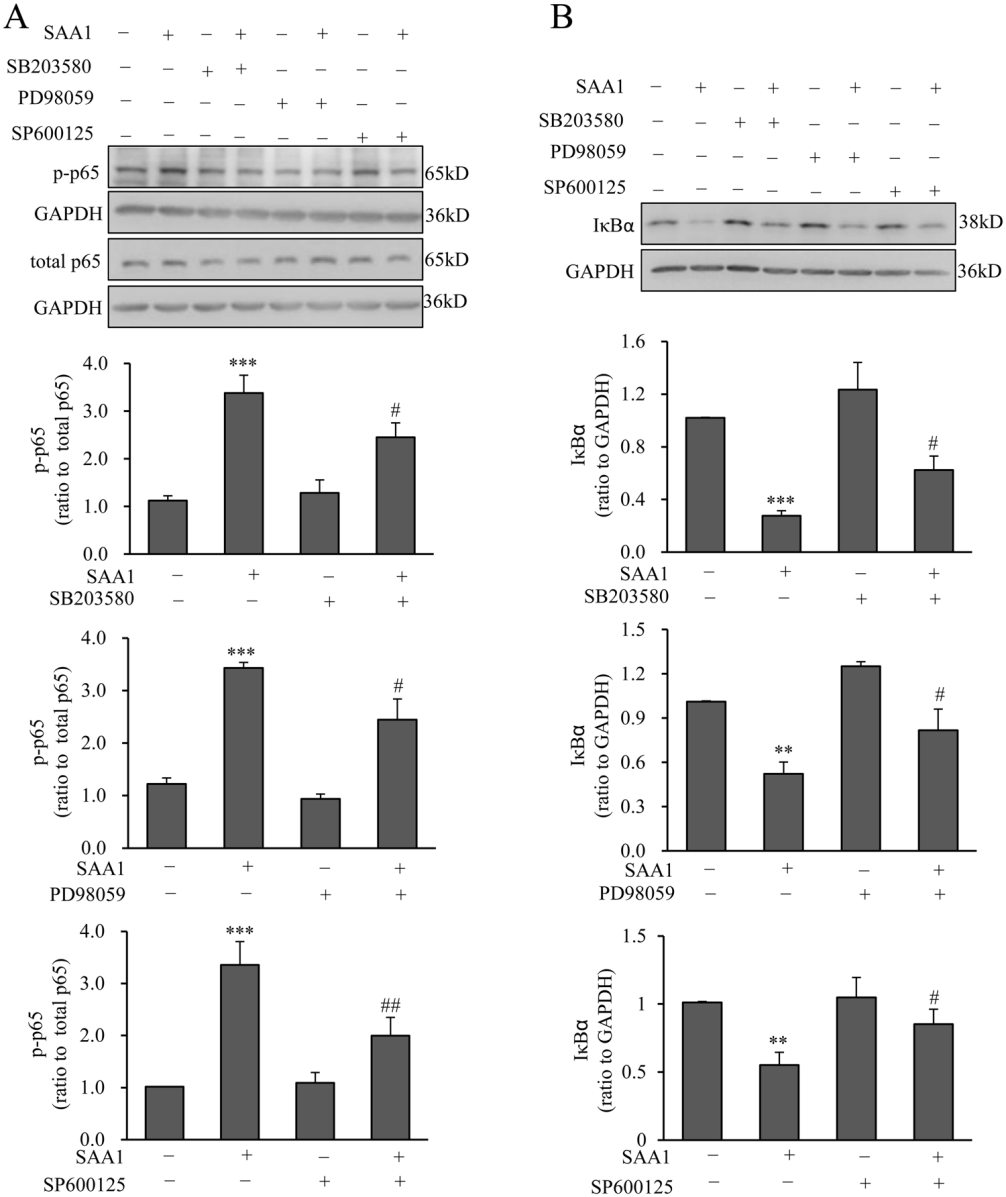
Effect of MAPK inhibitors on SAA1-induced increase in p65 phosphorylation and decrease in IκBα abundance in human amnion fibroblasts. Effect of p38 inhibitor SB203580 (10 µM), ERK1/2 inhibitor PD98059 (20 µM) and JNK inhibitor SP600125 (20 µM) on SAA1 (10 ng/mL, 1 hour)-induced increase in p65 phosphorylation (**A**) and decrease in IκBα abundance (**B**) in human amnion fibroblasts. n = 5. Top panels are the representative immunoblots. Data are the means ± SEM. Statistical analysis was performed with one-way ANOVA test. **P < 0.01, ***P < 0.001 vs. control (0), ^#^P < 0.05, ^##^P < 0.01 vs. SAA1.

Treatment of amnion fibroblasts with SAA1 (10 ng/mL; 15, 30, 60, 120 and 240 min) increased the phosphorylation of p38 (Fig. 3A), ERK1/2 (Fig. 3B) and JNK (Fig. 3C), the three members of MAPK family, in a time-dependent manner with maximal effects observed at 120 min for p38 and at 60 min for both ERK1/2 and JNK (Fig. 3A–C). Meanwhile, SAA1 (10 ng/mL; 15, 30, 60, 120 and 240 min) also increased the phosphorylation of p65 (Fig. 3D), a subunit of NF-κB complex, whereas decreased IκBα abundance (Fig. 3E), the inhibitor of NF-κB, with maximal effects observed at 60 min for both p65 phosphorylation and IκBα. The increase in p65 phosphorylation and decrease in IκBα by SAA1 could be blocked in part by inhibitors for p38, ERK1/2 and JNK respectively (Fig. 4A and B). To test whether these effects of SAA1 are due to the trace amount of endotoxin contained in the recombinant protein preparation, the cells were also treated with 5 pg/mL endotoxin and none of the effects of SAA1 described above was observed with endotoxin (Supplementary Figure S2C). These results suggest that SAA1 can activate not only the NF-κB pathway but also the MAPK pathway in amnion fibroblasts, and cross-talks between these pathways may exist in amnion fibroblasts.

**Figure 5:**
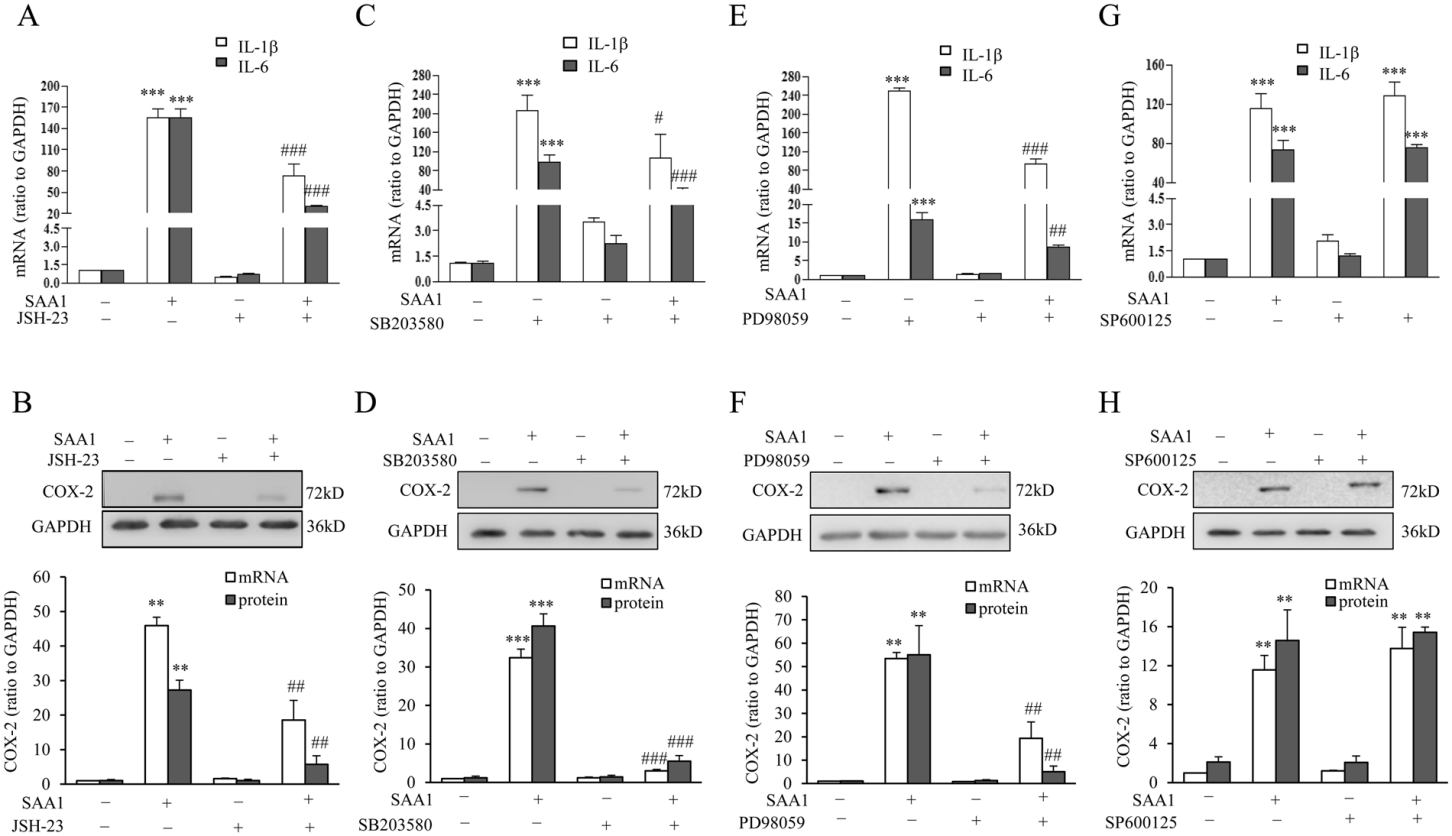
Role of NF-κB, p38, ERK1/2 and JNK in the induction of IL-1β, IL-6 and COX-2 by SAA1 in human amnion fibroblasts. (**A** and **B**) Effect of NF-κB inhibitor JSH-23 (10 µM) on SAA1 (10 ng/mL, 24 hours)-induced changes in IL-1β, IL-6 mRNA (**A**, n = 4), COX-2 mRNA and protein (**B**, n = 3) in human amnion fibroblasts. (**C** and **D**) The effect of p38 inhibitor SB203580 (10 µM) on SAA1 (10 ng/mL, 24 hours)-induced IL-1β, IL-6 mRNA (**C**, n = 4), COX-2 mRNA and protein (**D**, n = 3) in human amnion fibroblasts. (**E** and **F**) Effect of ERK1/2 inhibitor PD98059 (20 µM) on SAA1 (10 ng/mL, 24 hours)-induced IL-1β, IL-6 mRNA (E, n = 4), COX-2 mRNA and protein (**F**, n = 3) in human amnion fibroblasts. (**G** and **H**) Effect of JNK inhibitor SP600125 (20 µM) on SAA1 (10 ng/mL, 24 hours)-induced IL-1β, IL-6 mRNA (**G** n = 4), COX-2 mRNA and protein (**H**) n = 3) in human amnion fibroblasts. Top panels of (**B,D,F** and **H**) are the representative immunoblots. Data are the means ± SEM. Statistical analysis was performed with one-way ANOVA test. **P < 0.01, ***P < 0.001 vs. control (0), ^#^P < 0.05, ^##^P < 0.01, ^###^P < 0.001 vs. SAA1.

Further investigation demonstrated that the induction of IL-1β and IL-6 mRNA and COX-2 mRNA and protein by SAA1 (10 ng/mL) was significantly attenuated by NF-κB inhibitor JSH-23 (10 µM, Fig. 5A and B), p38 inhibitor SB203580 (10 µM, Fig. 5C and D) and ERK1/2 inhibitor PD98059 (20 µM, Fig. 5E and F) but not by JNK inhibitor SP600125 (20 µM, Fig. 5G and H) despite the observations that SAA1 phosphorylated JNK and inhibition of JNK partially blocked p65 phosphorylation by SAA1. These results suggest that SAA1 induced IL-1β, IL-6 and COX-2 expression through NF-κB, ERK1/2 and p38 MAPK-dependent pathways. Activation of JNK may elicit an intricate signaling network in addition to the effects observed in this study, which may jeopardize the effects of SAA1 on the expression of these pro-inflammatory factors in amnion fibroblasts.

### Role of TLR4 in the induction of IL-1β, IL-6 and COX-2 expression by SAA1 in human amnion fibroblasts

**Figure 6:**
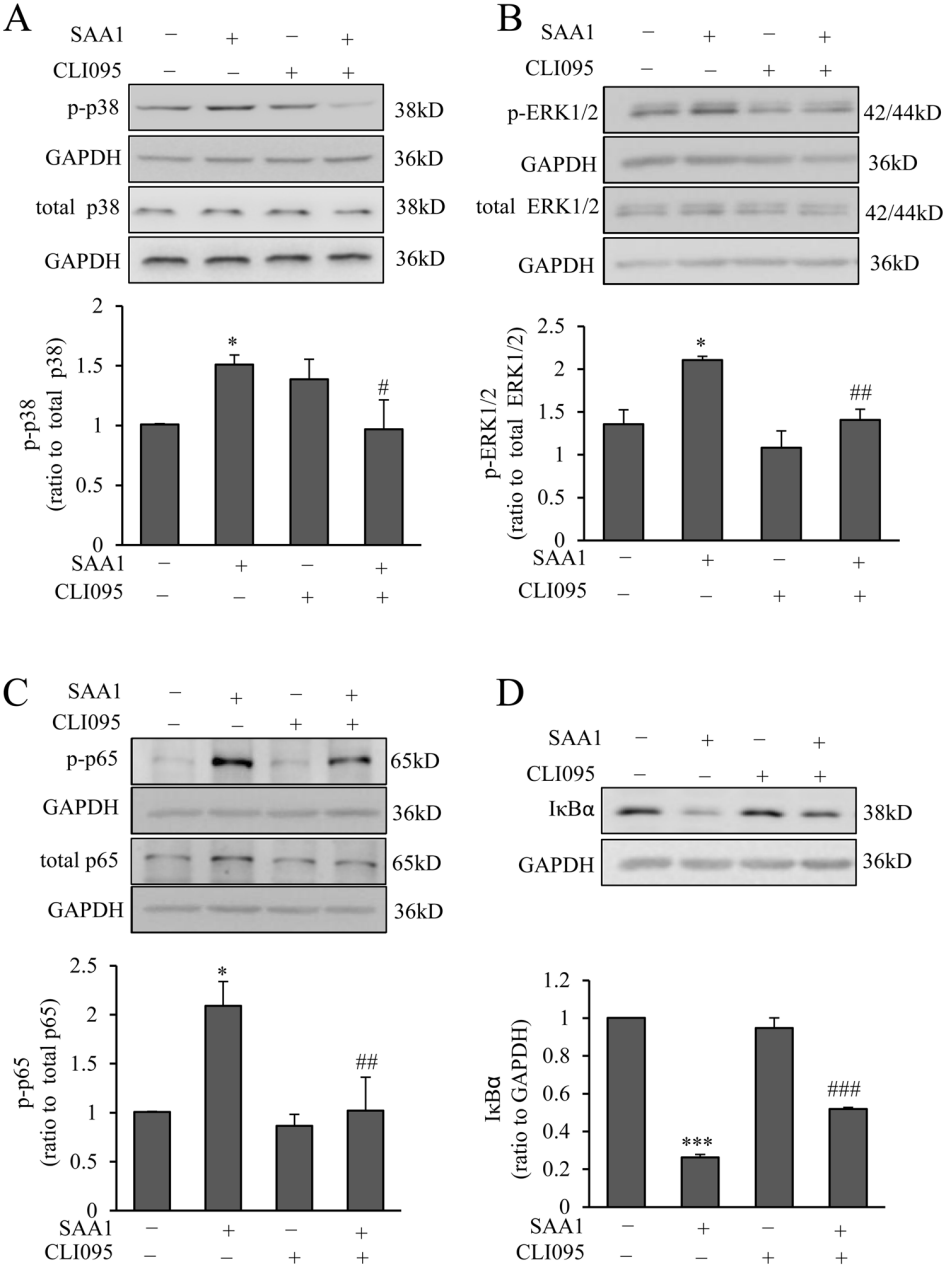
Role of TLR4 in the activation of MAPKs and NF-κB pathways by SAA1 in human amnion fibroblasts. Effect of TLR4 inhibitor CLI095 (5 μM) on the increases in the phosphorylation of p38 (**A**, n = 5), ERK1/2 MAPKs (**B**, n = 4) and p65 (**C**, n = 4), and the decrease in IκBα (**D**, n = 4) abundance induced by SAA1 (10 ng/mL, 60 min) in human amnion fibroblasts. Top panels are the representative immunoblots. Data are the means ± SEM. Statistical analysis was performed with one-way ANOVA test. *P < 0.05, ***P < 0.001 vs. control (0), ^#^P < 0.05, ^##^P < 0.01, ^###^P < 0.01 vs. SAA1.

**Figure 7:**
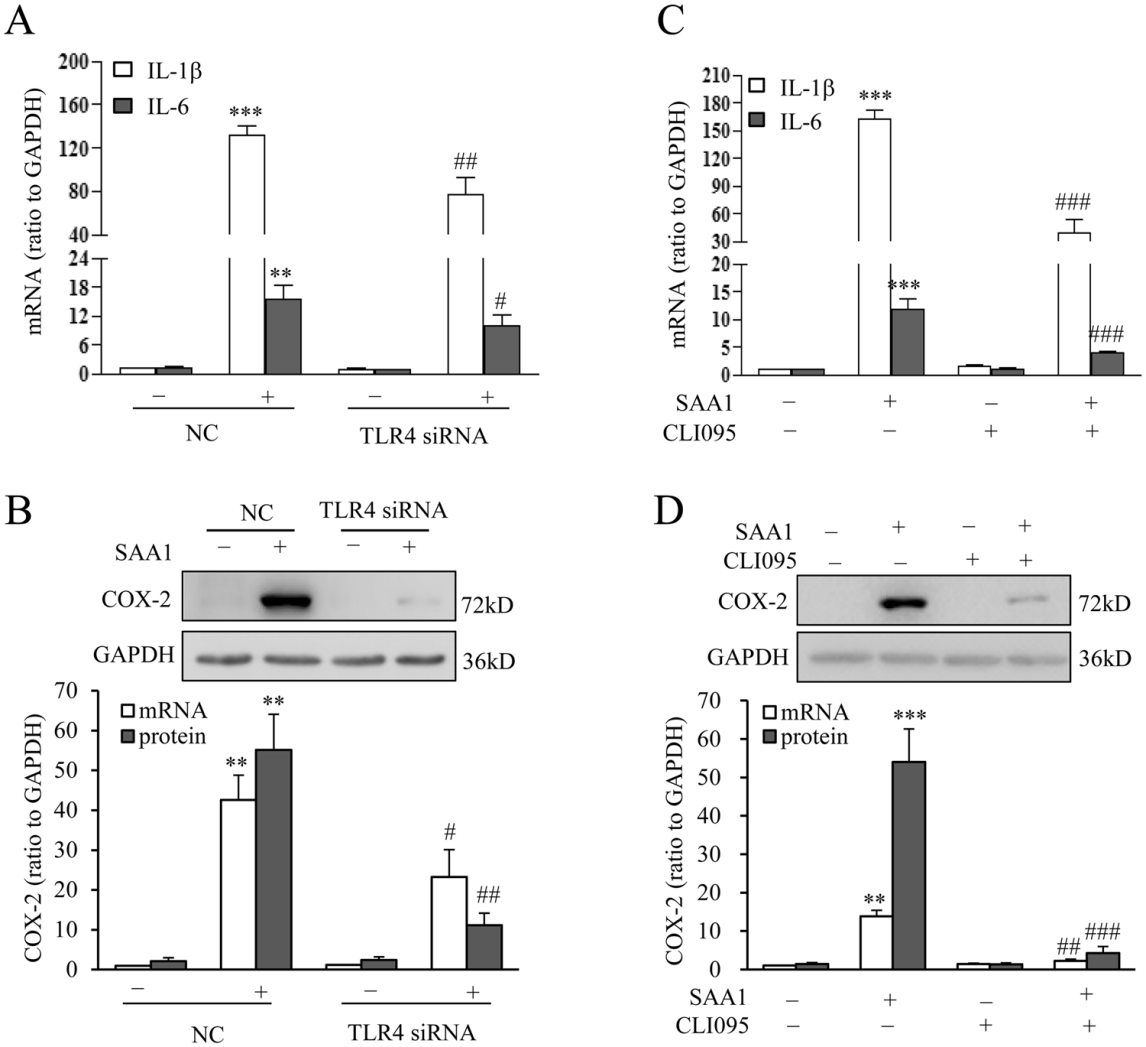
Role of TLR4 in the induction of IL-1β, IL-6 and COX-2 expression by SAA1 in human amnion fibroblasts. (**A**) Effect of siRNA-mediated knock-down of TLR4 on SAA1 (10 ng/mL, 24 hours)-induced changes in IL-1β, IL-6 mRNA (**A**, n = 4) and COX-2 mRNA and protein (**B**, n = 3) expression in human amnion fibroblasts. (**B**) Effect of TLR4 inhibitor on SAA1 (10 ng/mL, 24 hours)-induced changes in IL-1β, IL-6 mRNA (**C**, n = 4) and COX-2 mRNA and protein (**D**, n = 3) expression in human amnion fibroblasts. Top panels are the representative immunoblots. Data are the means ± SEM. Statistical analysis was performed with one-way ANOVA test. **P < 0.01, ***P < 0.001 vs. control (0) or NC, ^#^P < 0.05, ^##^P < 0.01, ^###^P < 0.001vs. SAA1 or NC + SAA1.

TLR4 inhibitor CLI095 (5 μM) significantly attenuated the increases in the phosphorylation of p65, ERK1/2 and p38 MAPKs, and the decrease in IκBα abundance induced by SAA1 (10 ng/mL, 60 min) in amnion fibroblasts (Fig. 6A–D). Consistently, CLI095 (5 μM) or siRNA-mediated knock-down of TRL4 also attenuated SAA1 (10 ng/mL, 24 h)-induced IL-1β, IL-6 and COX-2 expression in amnion fibroblasts (Fig. 7A–D). These results suggest that the induction of IL-1β, IL-6 and COX-2 expression by SAA1 via activation of the NF-κB, ERK1/2 and p38 MAPK pathways is mediated at least in part by TLR4 in amnion fibroblasts.

### Changes of SAA1 abundance following labor in human amnion tissue

**Figure 8:**
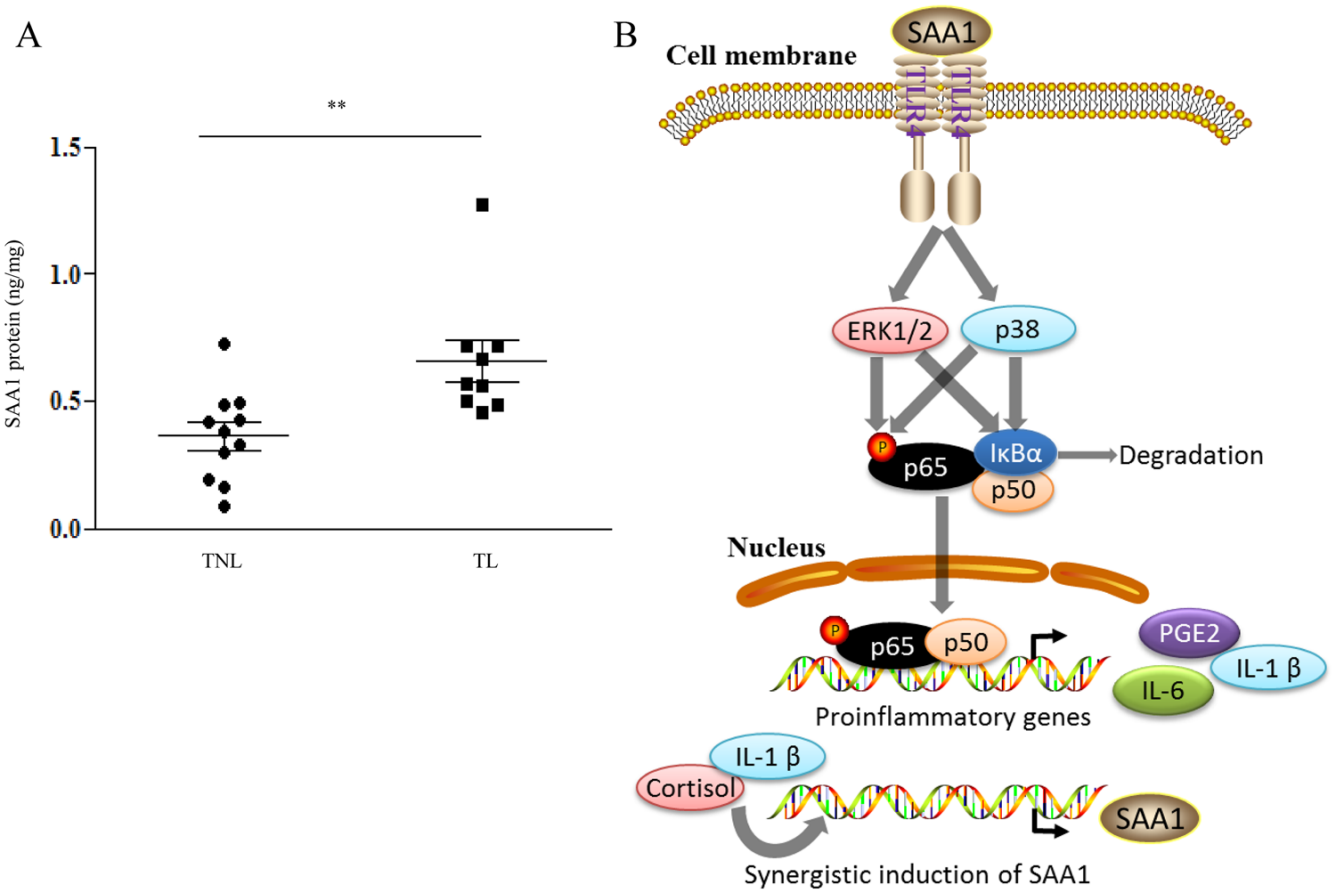
(**A**) Changes of SAA1 protein abundance in the amnion tissue following spontaneous labor. Abundance of SAA1 in human amnion tissue collected at term after spontaneous labor (TL) (n = 9) and after elective Cesarean section without labor (TNL) (n = 11). Data are the means ± SEM. Statistical analysis was performed with un-paired Student’s t-test. **P < 0.01 or TNL. (**B**) Signaling pathways uncovered in this study underpinning the effects of SAA1 on the expression of proinflammatory genes in human amnion fibroblasts. By binding to TLR4, SAA1 activates ERK1/2 and p38, which increase not only the phosphorylation of p65 but also the degradation of IκB resulting in the activation of NFκB. Activated NFκB induces the expression of proinflammatory genes including IL-1β, IL-6 as well as COX-2, the rate-limiting enzyme in PGE2 synthesis. In turn, IL-1β stimulates the expression of SAA1 synergistically with cortisol, a biologically active glucocorticoid regenerated by 11β-hydroxysteroid dehydrogenase 1 (11β-HSD1) from cortisone in human fetal membranes.

Enzyme immunoassays showed that SAA1 protein abundance was significantly increased in the amnion tissue obtained from spontaneous deliveries as compared with those from elective c sections without labor (Fig. 8A).

## Discussion

We have demonstrated in this study that SAA1 is expressed in the amnion and chorion of human fetal membranes. The amnion fibroblasts are capable of secreting SAA1, and the secretion can be augmented by either IL-1β or cortisol alone and by IL-1β and cortisol synergistically. SAA1 is able to stimulate the production of proinflammatory cytokines such as IL-1β and IL-6 as well as the uterotonic hormone PGE2, via TLR4-mediated activation of p38, ERK1/2 and NF-κB pathways in human amnion fibroblasts (Fig. 8B). As the concentrations of SAA1 used in this study fall well within the range detected in both the culture medium from cultured amnion fibroblasts and freshly homogenized amnion tissues, the effects of SAA1 observed in this study are highly relevant to *in vivo* situations. Since the abundance of SAA1 is increased significantly in the amnion tissue following labor, we believe that the production of SAA1 by amnion fibroblasts may participate in human parturition by stimulating the production of inflammation mediators in the amnion tissue. Although the effect of labor on SAA1 abundance can be either a cause or effect of labor, we believe that the increases in SAA1 abundance in the amnion tissue occur before labor and are further enhanced during labor since the expression of SAA1 is under the up-regulation of glucocorticoids, and it is known that the regeneration of cortisol by 11β-hydroxysteroid dehydrogenase 1 (11β-HSD1) is increasing with gestational age^32^ and is further exaggerated during labor in the amnion^28, 33, 34^.

In humans, there are four SAA genes (SAA1, SAA2, SAA3 and SAA4)^35, 36^. SAA1 and SAA2 code for the inducible forms of SAA proteins^3, 37^, and SAA4 encodes a constitutively expressed SAA protein, while SAA3 is a pseudogene^38^. SAA1 is so far the best characterized SAA member and the expression of SAA1 has been documented in various tissues in human body with the liver as the dominant source of plasma SAA1 during the acute-phase of an inflammatory response^39^. Although SAA1 has been studied in tissues in the non-pregnant state, only a few studies have focused on SAA1 in pregnancy. In pregnancy, SAA1 transcript has been demonstrated to be present in the villous trophoblasts and in trophoblast-derived cell lines^20, 40^. In early pregnancy, SAA1 produced in the trophoblasts is demonstrated to be associated with the invasion of extravillous trophoblasts^20^. To the best of our knowledge, this study has demonstrated for the first time that human fetal membranes are capable of synthesizing and secreting SAA1. Because SAA1 is capable of stimulating the production of not only pro-inflammatory cytokines such as IL-1β and IL-6, but also PGE2, the well-recognized potent uterotonic mediator of labor^23, 27^, SAA1 is possibly an important factor produced by the amnion fibroblasts in human parturition. Since SAA1 is a factor released during the acute phase response to infection and other stress challenges, the actions of SAA1 revealed in this study are probably among the earliest events that initiate both normal and pathogen-induced preterm birth.

In a study investigating the changes of plasma SAA1 during and after human labor, a constant level was observed during labor regardless of vaginal delivery or caesarean section^41^. However, a constant plasma concentration does not necessarily mean no dynamic changes of SAA1 concentration within the intrauterine tissues prior to labor or during labor. Moreover, in a LPS-induced mouse model of preterm birth, significant increases in SAA2 levels were observed in the plasma of LPS-treated animals^42^, which indicates a possible role of SAA proteins in parturition. This study demonstrated that SAA1 was increased in the amnion tissue with normal labor. A further increase in the abundance of SAA1 in the amnion is expected in the presence of infection for the reason that the proinflammatory cytokines such as IL-1β could stimulate SAA1 production in cultured amnion fibroblasts. Since IL-1β and SAA1 enhance each other’s production, we propose that a feed-forward mechanism in terms of IL-1β and SAA1 synthesis may exist in the amnion in both normal and infection-induced labor. These effects of SAA1 on the production of proinflammatory cytokines and prostaglandins in the amnion further suggest that the increases in SAA1 in the amnion tissue may be associated with the initiation of labor.

Toll-like receptors are known to mediate the primary responses to infection. Among the Toll-like receptors, TLR2 and TRL4 have been reported to mediate the effects of SAA1 in various cell systems^13, 14, 43–45^. In a recent study by Ebert *et al*.^46^, SAA1 was shown to stimulate a pro-inflammatory phenotype via TLR4 in mesenchymal stem cells by activation of p38 and NFκB pathways, which was believed to be part of the early phase of osteogenic differentiation and the development of senescence. Here we demonstrated that similar signaling pathways underpinned the actions of SAA1 in human amnion fibroblasts. In addition to the activation of p38, we found that SAA1 is also capable of activating ERK1/2 and JNK in amnion fibroblasts. However, despite of the activation of JNK, we found that the effects of SAA1 on the expression of IL-1β, IL-6 and COX-2 were dependent on p38, ERK1/2 and NF-κB activation but not on JNK activation. Moreover, this study also revealed that SAA1 can activate NF-κB via breakdown of IκBα and phosphorylation of p65, which may be mediated at least in part by p38, ERK1/2 and JNK activation, since the degradation of IκBα and phosphorylation of p65 by SAA1 were partially inhibited by inhibitors for p38, ERK1/2 and JNK respectively. Our findings are consistent with a recent published work showing that SAA1 is capable of activating IκBα kinase which then phosphorylates IκBα resulting in its degradation in hepatic stellate cells. SAA1 also activated JNK, ERK1/2 and NF-κB in these cells^16^. Since the effects of SAA1 can be blocked by either TLR4 antagonist or siRNA-mediated knock-down of TLR4 to some extent, we believe that TLR4 mediates at least in part the effects of SAA1 in amnion fibroblasts. However, at present we cannot rule out the roles of other receptors such as TLR2 and FPR2 in mediating of the effects of SAA1 in this cell type. At present, we are also unclear as to why blockade of the JNK pathway is ineffective in the inhibition of the effects of SAA1 on the expression of these pro-inflammatory factors in the presence of JNK’s participation in the activation of NF-κB by SAA1. An intricate signaling network might be associated with JNK activation. In addition to the phosphorylation of p65, JNK is also capable of phosphorylating a number of other transcription factors^47^, which may offset the effects of NF-κB activation by SAA1.

In conclusion, this study has demonstrated for the first time that SAA1 can be produced by human amnion and its abundance is increased significantly in the amnion following labor possibly under the synergistic drive of increased cortisol regeneration and proinflammatory cytokine production in the amnion. Since SAA1 is capable of stimulating the production of labor-initiating factors such as IL-1β, IL-6 and PGE2 by amnion fibroblasts, we believe that the increased abundance of SAA1 may be associated with the initiation of human parturition.

## Methods

### Human fetal membrane collection

Human fetal membranes were obtained from uncomplicated term (37–40 weeks) pregnancies after elective cesarean section without labor (term non-labor, TNL) and after spontaneous labor (term labor, TL) with written informed consents from patients under a protocol approved by the Ethics Committee of Ren Ji Hospital, School of Medicine, Shanghai Jiao Tong University. All methods were performed in accordance with the relevant guidelines and regulations. Pregnancies with complications such as preeclampsia, fetal growth restriction, gestational diabetes and chorioamnionitis were excluded from this study. The fetal membranes obtained from TNL (n = 3) were used for immunohistochemical examination of SAA1 localization. The amnion layer was peeled from the fetal membranes collected from TNL (n = 38) for the isolation of amnion fibroblasts. The amnion tissues separated from the fetal membranes obtained from both TNL (n = 11) and TL (n = 9) were used for analysis of SAA1 changes with labor.

### Immunohistochemical staining of SAA1 in human fetal membranes and amnion fibroblasts

Tissue sections were cut from paraffin-embedded fetal membranes collected from TNL after fixing in 4% paraformaldehyde, and amnion fibroblasts were fixed in 4% paraformaldehyde 3 days after plating. The methods for amnion fibroblast isolation and culture are described below. Amnion tissue sections and fibroblasts were permeabilized with 0.2% Triton X-100 before primary antibody application. Following blocking, an antibody derived from the rabbit against human SAA1 (Abcam, Cambridge, UK) was applied at 1:100 dilutions for overnight incubation at 4C. For tissue sections, an anti-rabbit IgG antibody conjugated with biotinylated horseradish peroxidase H was applied as the secondary antibody and the red color reaction was developed using substrate 3-amino-9-ethyl carbazole (Vector Laboratories, Burlingame, CA). For cultured cells, in addition to SAA1, a mouse antibody against vimentin, a mesenchymal cell marker, was also applied at 1:100 (Proteintech, Wuhan, China) for a double staining procedure. Alexa Fluor 488 (green color)-labeled anti-mouse IgG and Alexa Fluor 594 (red color)-labeled anti-rabbit IgG (Proteintech) were used as the secondary antibodies. Nuclei were counterstained with DAPI (blue color). The stained slides were examined using either a regular light microscope or a fluorescence microscope (Zeiss, Germany). To test the specificity of the immunostaining, separate slides were exposed to preimmune serum instead of the primary antibodies.

### Amnion fibroblast culture

Amnion fibroblasts were isolated from the amnion collected from TNL as described previously^28^. Briefly, the amnion was peeled from the membranes and the amnion tissue was digested with 0.125% trypsin (Life Technologies Inc., Grand Island, NY) and then washed thoroughly with normal saline to remove epithelial cells. The remaining tissue was digested with 0.1% collagenase (Sigma, St. Louis, MO) and the fibroblast cells in the digestion medium were collected by centrifugation. The fibroblasts were cultured at 37 °C in 5% CO_2_–95% air in DMEM containing 10% FBS plus 1% antibiotics (all from Life Technologies Inc.) for 3 days before treatments. The identity of cells was verified by staining for vimentin and more than 95% of the cells were positive for vimentin.

### Treatment of amnion fibroblasts

Three days after plating, the amnion fibroblasts were treated in the culture medium free of phenol red and FBS. To investigate the basal, cortisol and IL-1β-induced expression of SAA1, the cells were treated with or without cortisol (0.01, 0.1 and 1 µM), or IL-1β (1, 5 and 10 ng/mL), or combination of cortisol (1 µM) and IL-1β (10 ng/mL) for 24 hours. To study the effects of SAA1 on the activation of NF-κB and MAPKs, and on the abundance of IL-1β, IL-6 and COX-2, the cells were treated with recombinant human apo-SAA1 (5, 10 and 50 ng/mL, endotoxin < 0.1 ng/µg, PeproTech Inc., Rocky Hill, NJ) and then the phosphorylation of p65, a subunit of NF-κB, p38, ERK1/2 and JNK was determined. The treatment time is given in the respective figure legends. To test whether the effects of SAA1 were ascribed to the trace amount of endotoxin in the preparation of recombinant SAA1, the cells were treated with 5 pg/mL endotoxin, which is equivalent to the amount of endotoxin contained in 50 ng of SAA1 according to the manual provided by the manufacturer. To further test whether the effects of SAA1 were ascribed to SAA1 *per se* rather than comtaminating endoctoxin, the cells were treated with SAA1 (10 ng/mL) or lipopolysaccharides (LPS) (5 ng/mL, Sigma) in the presence of an endotoxin inhibitor polymyxin B (25 µg/mL, Sigma). To examine whether MAPKs were involved in the phosphorylation of p65 and degradation of IκBα, the cells were treated with SAA1 (10 ng/mL) in the presence or absence of p38 MAPK inhibitor SB203580 (10 µM; Selleck, Houston, TX), ERK1/2 inhibitor PD98059 (20 µM; Sellcek) and JNK inhibitor SP600125 (20 µM; Sigma) and then the abundance of IκBα and phosphorylated p65 was measured. To determine the involvement of NF-κB and MAPKs in the induction of IL-1β, IL-6 and COX-2 expression by SAA1, the fibroblasts were treated with SAA1 (10 ng/mL) for 24 hours in the presence or absence of p65 nuclear translocation inhibitor JSH-23 (10 µM; Sigma), SB203580 (10 µM), PD98059 (20 µM) and SP600125 (20 µM). To study the involvement of TLR4 in the activation of NF-κB and MAPKs, and in the induction of IL-1β, IL-6 and COX-2 expression, the cells were treated with SAA1 (10 ng/mL) in the presence or absence of TLR4 inhibitor CLI-095 (5 μM; Invivogen, San Diego, CA) or small interfering RNA (siRNA, Gene Pharma, Shanghai, China)-mediated knockdown of TLR4. The method of siRNA transfection is described below. After treatments, the culture media were collected for the measurements of SAA1, IL-1β and IL-6 with enzyme immunoassays (ELISA) or measurements of PGE2 with competitive enzyme immunoassays (EIA), and the cells were processed for the extraction of total RNA and cellular protein for analyses of SAA1, IL-1β, IL-6 or COX-2 mRNA or protein abundance with quantitative real time PCR (qRT-PCR) and western blotting respectively.

### siRNA transfection

To study the role of TLR4 in the mediation of SAA1-induced effects, amnion fibroblasts were transfected with 50 nM siRNA (GenePharma) against TLR4 (5′-CCCACAUUGAAACUCAAAUtt-3′) or randomly scrambled siRNA using an electroporator (Nepa Gene, Chiba, Japan) at 175 V for 5 ms following a protocol as described previously^28^. The cells were then incubated in DMEM containing 10% FBS and 1% antibiotics for three days before treatment. The efficiency of knockdown is shown in Supplementary Figure S1.

### Quantitative real time PCR

After treatments, total RNA was extracted and reverse-transcribed to cDNA using a commercial kit (TaKaRa, Dalian, China). The amounts of IL-1β, IL-6 and COX-2 mRNA were determined with qRT-PCR using the above transcribed cDNA and power SYBR® Premix Ex Taq^TM^ (TaKaRa) following a protocol described previously^28^. The absolute mRNA abundance in each sample was calculated according to a standard curve set up using serial dilutions of known amounts of specific templates against corresponding cycle threshold values. The housekeeping gene, GAPDH, was amplified in parallel as an internal control. The primer sequences used for qRT-PCR are illustrated in Supplementary Table S1.

### Western blotting

After treatments, the cells were lysed in ice-cold radio immune-precipitation assay (RIPA) lysis buffer (Active Motif, Carlsbad, CA) containing a protease inhibitor cocktail (Sigma) and a phosphatase inhibitor (Active Motif). After determination of protein concentration, the extracted protein was analyzed with western blotting following a standard protocol as described previously^28^. The primary antibodies against p65 (1:1000, Cell Signaling, Danvers, MA), phosphorylated p65 at Ser 536 (1:1000, Cell Signaling), p38 (1:500, Cell Signaling), phosphorylated p38 at Thr180/Tyr182 (1:200, Cell Signaling), ERK1/2 (1:200, Cell signaling), phosphorylated ERK1/2 at Thr202/Tyr204 (1:200, Cell signaling), JNK (1:1000, Cell signaling), phosphorylated JNK at Thr183/Tyr185 (1:1000, Cell signaling), IκBα (1:200, Santa Cruz, Santa Cruz, CA) and COX-2 (1:200, Santa Cruz) were used to probe the respective target proteins, which were followed by incubations with secondary antibodies and an enhanced chemiluminescent detection system (Millipore, Billerica, MA). Internal loading controls were probed with a GAPDH antibody (1:10000, Proteintech).

### Measurements of SAA1, IL-1β, IL-6 and PGE2

The basal and IL-1β-induced SAA1 secretions were determined by measuring SAA1 abundance in the culture medium with an ELISA kit (R&D Systems, Minneapolis, MN). After treatment with SAA1 (10 ng/mL, 24 hours), IL-1β and IL-6 in the culture medium were measured with ELISA kits (R&D systems) and PGE2 in the culture medium was measured with EIA kit (Cayman, Ann Arbor, MI) according to the protocols provided by manufacturers.

To compare the amounts of SAA1 in the amnion tissue with or without labor, tissue pieces were cut from the amnion 5 cm within the rupture site and then ground in liquid nitrogen. The ground tissue was homogenized and lysed in ice-cold RIPA lysis buffer containing a protease inhibitor cocktail and centrifuged. Protein in the supernatant was collected for SAA1 protein analysis with an ELISA kit (R&D Systems) following the protocol provided by the manufacturer.

### Statistical analysis

All data are reported as means + SEM. The number of each study represents separate experiments using amnion from different pregnancies. After examination for normal distribution, statistical analysis was performed with paired or unpaired Student’s t-test or one-way ANOVA test followed by the Newman-Keuls multiple comparison test where appropriate. Significance was set at P < 0.05.

## Electronic supplementary material

supplementary figures and table
