# Supplementary material for: Induction of pro-inflammatory genes by serum amyloid A1 in human amnion fibroblasts
Source: Sci Rep. 2017 Apr 6;7:693. doi: 10.1038/s41598-017-00782-9 (PMC5429602; doi:10.1038/s41598-017-00782-9)
Supplement: Supplementary file 1 — supplementary figures and table [file 41598_2017_782_MOESM1_ESM.pdf]

Induction of pro-inflammatory genes by serum amyloid A1 in human amnion fibroblasts

Wenjiao Li[1,2+], Wangsheng Wang[1,2+], Rujuan Zuo[1,2], Chao Liu[1,2], Qun Shu[3], Hao Ying[4], Kang Sun[1,2*]

[1]Center for Reproductive Medicine, Ren Ji Hospital, School of Medicine, Shanghai Jiao Tong University. Shanghai, P.R. China.
[2]Shanghai Key Laboratory for Assisted Reproduction and Reproductive Genetics, Shanghai, P.R. China.
[3]Department of Obstetrics and Gynecology, Zhongshan Hospital, Fudan University, Shanghai, P.R. China.
[4]Shanghai First Maternity and Infant Hospital, Tongji University School of Medicine, Shanghai, P.R. China

Supplementary figures and table

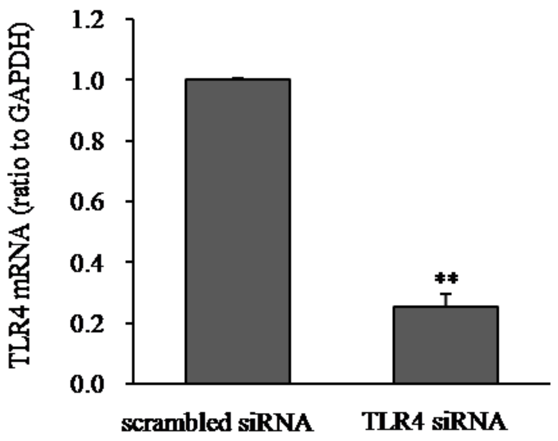

Supplementary Figure S1 Efficiency of siRNA-mediated knock-down of TLR4 in human amnion fibroblasts. Data are the mean ± SEM. n = 4. Statistical analysis was performed with paired Student's t test. **P<0.01 *vs* scrambled siRNA.

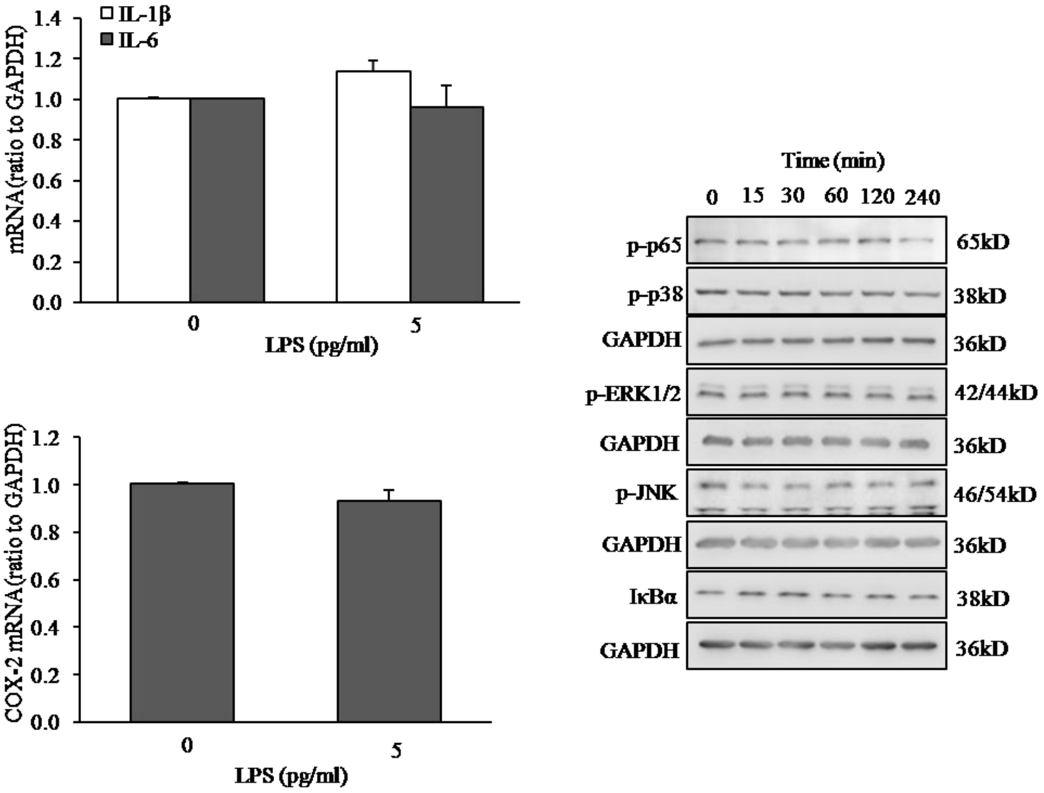

Supplementary Figure S2 (A and B) LPS (5 pg/ml, 24 hours) had no effect on the expression of IL-1β, IL-6 and COX-2 in human amnion fibroblasts. Data are the mean ± SEM. n=3. **(C)** LPS (5 pg/ml) had no effect on either the abundance of phosphorylated p65, p38, ERK1/2 and JNK or the abundance of IκBα.

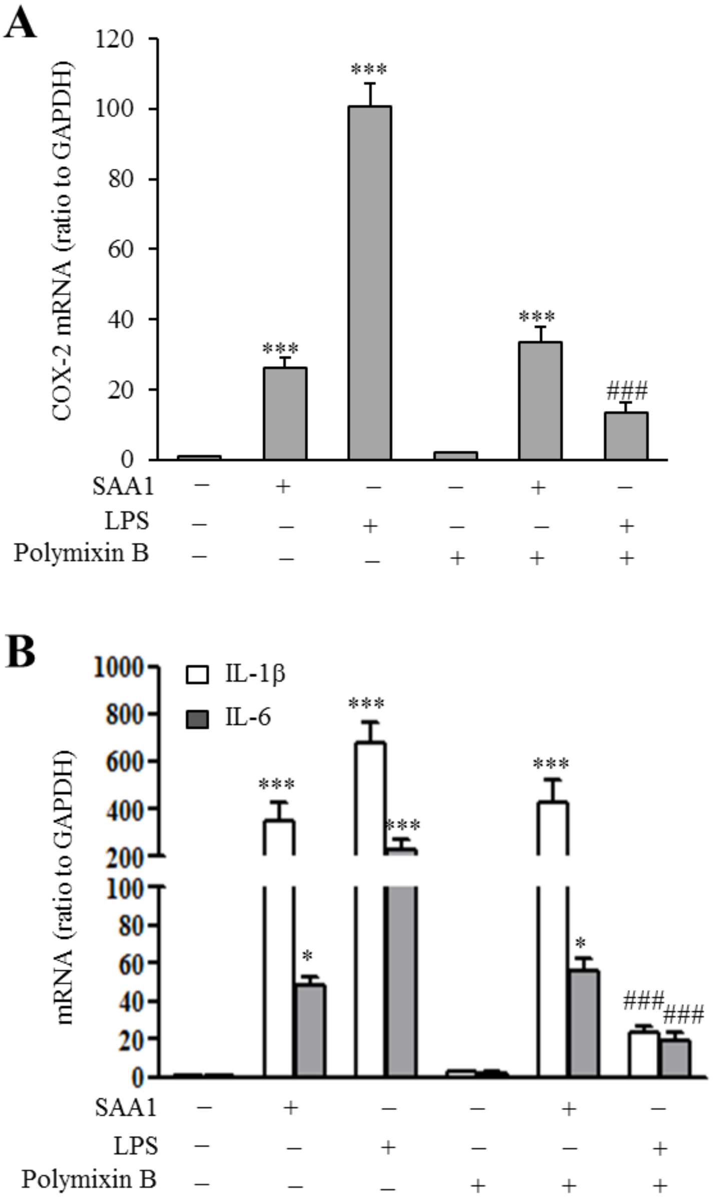

Supplementary Figure S3 (A and B) LPS inhibitor Polymixin B (25 μg/mL, 24 hours) had no effect on SAA1 (10 ng/mL)-induced but blocked LPS (5 ng/mL)-induced expression of COX-2 (A), IL-1β and IL-6 (B) in human amnion fibroblasts. Data are the mean ± SEM. n = 3. *P<0.05, ***P<0.001 *vs* control (0); ###P<0.001 vs LPS.

Supplementary Table S1 Primer sequences used for qRT-PCR

Genes	Primers sequence
IL1B	Forward: 5'-CCACCTCCAGGGACAGGATA -3'
	Reverse: 5'-AACACGCAGGACAGGTACAG -3'
IL6	Forward: 5'-CTTCGGTCCAGTTGCCTTCT -3'
	Reverse: 5'-GTGCCTCTTTGCTGCTTTCA -3'
PTGS2	Forward: 5'-TGTGCAACACTTGAGTGGCT -3'
	Reverse: 5'-ACTTTCTGTACTGCGGGTG -3'
TLR4	Forward: 5'-AAAATCCCCGACAACCTCCC-3'
	Reverse: 5'-AGAGGTGGCTTAGGCTCTGA-3'
GAPDH	Forward: 5'-CCCCTCTGCTGATGCCCCCA-3'
	Reverse: 5'-TGACCTTGGCCAGGGGTGCT-3'
